# A Matrix-Variate *t* Model for Networks

**DOI:** 10.3389/frai.2021.674166

**Published:** 2021-05-13

**Authors:** Monica Billio, Roberto Casarin, Michele Costola, Matteo Iacopini

**Affiliations:** ^1^Department of Economics, Ca' Foscari University of Venice, Venice, Italy; ^2^Department of Econometrics and Data Science, Vrije Universiteit Amsterdam, Amsterdam, Netherlands; ^3^Tinbergen Institute, Amsterdam, Netherlands

**Keywords:** Bayesian, financial markets, matrix-variate distributions, networks, *t* distribution, C11, C32, C58, 62F15, 62M10, 65C05

## Abstract

Networks represent a useful tool to describe relationships among financial firms and network analysis has been extensively used in recent years to study financial connectedness. An aspect, which is often neglected, is that network observations come with errors from different sources, such as estimation and measurement errors, thus a proper statistical treatment of the data is needed before network analysis can be performed. We show that node centrality measures can be heavily affected by random errors and propose a flexible model based on the matrix-variate *t* distribution and a Bayesian inference procedure to de-noise the data. We provide an application to a network among European financial institutions.

## 1. Introduction

A network can be defined as a set of nodes and edges, which represent a relationship among the nodes (Newman et al., 2006; Newman, 2018). A wide spectrum of relational, spatial, and multivariate data from many fields, such as sociology, biology, environmental, and neuroscience, admits a natural representation as a network. In mathematical terms, a network can be represented through the notion of a graph and its properties. For an introduction to graph theory and random graphs, we refer the interested reader to Bollobás (1998) and Bollobás (2001). See Jackson (2008) for an introduction to network theory in social sciences. In this paper, we will use the two terms interchangeably. As an example, in financial networks, a node represents a firm and an edge has the interpretation of a financial relationship between two firms.

In finance, the extraction of unobserved networks from time series data has attracted the attention of many researchers since the recent financial crisis (e.g., Billio et al., 2012; Diebold and Yılmaz, 2014). A large number of different methodologies have been proposed for the estimation of financial networks from firm return series (e.g., Barigozzi and Brownlees, 2019; Bräuning and Koopman, 2020), in particular in the Bayesian approach. For example, Billio et al. (2019) propose a Bayesian non-parametric Lasso prior distribution for vector autoregressive (VAR) models, which provides a sparse estimation of the VAR coefficients and classifies the non-zero coefficients into different clusters. They extract causal networks among financial assets and find that the resulting network topologies match the features of many real-world networks. Ahelegbey et al. (2016a,b) exploit graphical models to specify both the contemporaneous and the lagged causal structures in Bayesian VAR models. In a related contribution, Bianchi et al. (2019) investigate the temporal evolution in systemic risk using a Markov-switching graphical SUR model.

The inferred network structure is intrinsically contaminated by a certain degree of estimation error, which may cumulate with other sources of errors, such as model misspecification and measurement error. Consequently, the direct use of estimated networks as inputs in network analyses (e.g., Casarin et al., 2020; Wang et al., 2021) may result in misleading conclusions. This calls for the definition of suitable tools for cleaning the data from random disturbances, thus enabling to perform valid statistical analyses of the networks.

In this paper, we propose a new Bayesian model for network data with matrix-variate *t* errors which accounts for heavy tails (Tomarchio et al., 2020). The inferential procedure is based on data augmentation and conjugate prior distributions that allow for an efficient posterior sampling scheme. In addition to the studies on financial network extraction, our paper also contributes to the literature on matrix-variate models and financial connectedness.

Motivated by the increasing availability of large and multidimensional data, the use of matrix-variate distributions in time series econometrics has flourished during the last decade. The main domains where these models have been successfully applied include the classification of longitudinal datasets (Viroli, 2011), network analysis (Durante and Dunson, 2014; Zhu et al., 2017, 2019), factor analysis (Wang et al., 2019; Chen et al., 2020), stochastic volatility modeling (Gouriéroux et al., 2009; Golosnoy et al., 2012), and Gaussian dynamic linear modeling (Wang and West, 2009).

Finally, we aim to contribute to the financial economics literature by applying the proposed method for de-noising network data extracted from European firms' stock market returns. Then, we analyze the connectedness of the network and compare the results to those obtained from a direct analysis of the network raw data. Our simulation results provide an estimate of the bias in the network centrality measures induced by errors in the edges and show that the proposed approach is effective in correcting for the bias. Our empirical analysis confirms the presence of variability in the network edges. Furthermore, comparing network statistics between the raw network data and the filtered one, we find substantial evidence of differences in the most frequently used statistics, such as out-degree, eigenvector, betweenness, and closeness centrality measures.

The remaining of the paper is structured as follows: section 2 introduces a new linear model for matrix-valued data, then section 3 presents a Bayesian inference procedure. The results of an empirical analysis on real network data are illustrated in section 4. Finally, section 5 concludes.

## 2. A Matrix-Variate *t* Model

Let $G_{t}=\left(V,H_{t}\right)$, *t* = 1, …, *T*, be a sequence of networks (Boccaletti et al., 2014), where *H*_*t*_ ⊂ *V* × *V* is the edge set and *V* = {1, …, *n*} is the set of nodes. In our application, $G_{t}$ is a Granger network where the nodes represent institutions from different sectors and directed edges represent financial linkages. A directed edge from node *j* to node *i* represents a Granger-causal relationship from firm *j* to firm *i*, and is associated to the element *Y*_*ij, t*_ of the adjacency matrix *Y*_*t*_.

The connectivity structure of a *n*-dimensional network $G_{t}$ can be represented through a *n*-dimensional square matrix *Y*_*t*_, called the adjacency matrix. Each element *Y*_*uv, t*_ of the adjacency matrix is non-zero if there is an edge from institution *v* to institution *u* with *u, v* ∈ *V*, and 0 otherwise, where *u* ≠ *v*, since self-loops are not allowed.

Unfortunately, most frequently the connectivity structure among financial institutions is not directly observable, thus requiring suitable statistical tools to extract the latent network topologies that are characterized by estimation errors. For example, our data relies on a Granger causality approach to extract network observations from financial price series, which, in turn, may be contaminated by the presence of measurement noise. Overall, these multiple sources of errors may yield an imperfect observation of the true connectivity structure, calling for the adoption of a proper de-noising procedure before performing network analyses.

We propose a matrix-variate linear stochastic model to deal with measurement and estimation errors in the adjacency matrices. The noise process is assumed to follow a matrix-variate *t* distribution, that accounts for potentially large deviations of the observations from the mean. The proposed model is

(1) $$\begin{array}{r} Y_{t}=B+E_{t},\;E_{t}\overset{iid}{\sim }t_{n,n}\left(\nu ,0,\Sigma_{1},\Sigma_{2}\right),\;t=1,\ldots ,T, \end{array}$$

where *B* ∈ ℝ^*n* × *n*^ is a matrix of coefficients and $E_{t}\in {\mathbb{R}}^{n\times n}$ is a random error term. A random matrix *X* ∈ ℝ^*p* × *m*^ follows a matrix-variate *t* distribution, *X* ~ *t*_*p, m*_(ν, *M*, Σ_1_, Σ_2_), if it has probability density function

(2) $$\begin{array}{r} P\left(X|\nu ,M,\Sigma_{1},\Sigma_{2}\right)=\frac{\Gamma_{p}(\frac{\nu +m+p-1}{2})}{\pi^{\frac{mp}{2}}\Gamma_{p}(\frac{\nu +p-1}{2})}|\Sigma_{1}{|}^{-\frac{m}{2}}|\Sigma_{2}{|}^{-\frac{p}{2}}|I_{p}\\ +\Sigma_{1}^{-1}\left(X-M\right)\Sigma_{2}^{-1}{\left(X-M\right)}^{\prime }{|}^{-\frac{\nu +m+p-1}{2}} \end{array}$$

where Γ_*p*_(·) is the multivariate gamma function and |·| denotes the matrix determinant. The matrix *M* ∈ ℝ^*p* × *m*^ is the location parameter, ν > 0 is the degrees of freedom parameter, and the positive definite matrices $\Sigma_{1}\in {\mathbb{R}}^{p\times p}$ and $\Sigma_{2}\in {\mathbb{R}}^{m\times m}$ are scale parameters driving the covariances between each of the *p* rows and the *m* columns of *X*, respectively. For further details, see Chapter 4 in Gupta and Nagar (1999). Thanks to the properties of the matrix-variate *t* distribution, the unconditional mean and variance of *Y*_*t*_ are 𝔼(*Y*_*t*_) = *B*, if ν > 1, and 𝕍*ar*(*Y*_*t*_) = Σ_2_ ⊗ Σ_1_/(ν − 2), if ν > 2, where ⊗ denotes the Kronecker product.

## 3. Bayesian Inference

### 3.1. Prior Specification

In this section we describe the prior structure for the model parameters. For the coefficient matrix *B*, we assume a matrix normal distribution

(3) $$\begin{array}{l} B\sim N_{n,n}\left(0,\Omega_{1},\Omega_{2}\right), \end{array}$$

where Ω_1_ = ω_1_*I*_*n*_ and Ω_2_ = ω_2_*I*_*n*_, with ω_1_ > 0 and ω_2_ > 0 fixed. A random matrix *Z* ∈ ℝ^*p* × *m*^ follows a matrix normal distribution (Gupta and Nagar, 1999, Chapter 2), denoted by $X\sim N_{p,m}\left(M,\Sigma_{1},\Sigma_{2}\right)$, if its probability density function is

(4) $$\begin{array}{r} P\left(X|M,\Sigma_{1},\Sigma_{2}\right)={\left(2\pi \right)}^{-\frac{mp}{2}}|\Sigma_{1}{|}^{-\frac{p}{2}}|\Sigma_{2}{|}^{-\frac{m}{2}}\\ \exp(-\frac{1}{2}\mathrm{tr}(\Sigma_{2}^{-1}{\left(X-M\right)}^{\prime }\Sigma_{1}^{-1}\left(X-M\right))). \end{array}$$

The matrix normal distribution is equivalent to a multivariate normal distribution with a product-separable covariance structure, that is, $X\sim N_{p,m}\left(M,\Sigma_{1},\Sigma_{2}\right)$ is equivalent to $\mathrm{vec}\left(X\right)\sim N_{pq}\left(\mathrm{vec}\left(M\right),\Sigma_{2}⊗\Sigma_{1}\right)$, where vec(·) is a vectorization operator that stacks all the columns of a matrix into a column vector. Since Σ_2_ ⊗ Σ_1_ = (Σ_2_/*a*) ⊗ (*aΣ*_1_) for any *a* ≠ 0, the noise covariance matrices of the matrix-variate *t* distribution, Σ_1_, Σ_2_, are not identifiable and prior restrictions can be used to achieve identification. Nevertheless, in this paper we are interested in the variability of the errors as measured by the product Σ_2_ ⊗ Σ_1_, which is always identifiable. For the noise covariances, Σ_1_ and Σ_2_, we assume the following hierarchical prior distribution

(5) $$\begin{array}{r} \gamma \sim Ga\left(a_{\gamma },b_{\gamma }\right),\;\Sigma_{1}|\gamma \sim W_{n}\left(\gamma^{-1}{\text{Ψ}}_{1}^{-1},\kappa_{1}\right),\\ \Sigma_{2}|\gamma \sim {IW}_{n}\left(\gamma {\text{Ψ}}_{2},\kappa_{2}\right), \end{array}$$

where we use the shape-scale parametrization for the gamma distribution and the scale parametrization for the Wishart and inverse Wishart distributions, with densities

$$\begin{array}{l} P(\Sigma_{1}|\gamma^{-1}\Psi_{1}^{-1},\kappa_{1})=\frac{1}{2^{n\kappa_{1}/2}|\gamma^{-1}\Psi_{1}^{-1}{|}^{\kappa_{1}/2}\Gamma_{n}(\frac{\kappa_{1}}{2})}\\ \;|\Sigma_{1}{|}^{\frac{\kappa_{1}-n-1}{2}}\exp\left(-\frac{1}{2}\text{tr}(\gamma \Psi_{1}\Sigma_{1})\right)\\ \;P(\Sigma_{2}|\gamma \Psi_{2},\kappa_{2})=\frac{|\gamma \Psi_{2}{|}^{\kappa_{2}/2}}{2^{n\kappa_{2}/2}\Gamma_{n}(\frac{\kappa_{2}}{2})}\\ \;|\Sigma_{2}{|}^{-\frac{\kappa_{2}+n+1}{2}}\exp\left(-\frac{1}{2}\text{tr}(\gamma \Psi_{2}\Sigma_{2}^{-1})\right), \end{array}$$

where κ_1_ and κ_2_ are the degrees of freedom parameters and Γ_*n*_(·) is the multivariate gamma function (see Gelman et al., 2014, Appendix A, p. 577). The common scale γ allows for various degrees of prior dependence in the unconditional joint distribution of (Σ_1_, Σ_2_). Finally, since the object of interest is the mean of *Y*_*t*_, which is defined only for ν > 1, we assume the following gamma prior distribution truncated on the interval (1, +∞)

(6) $$\begin{array}{r} \nu \sim TGa\left(a_{\nu },b_{\nu };1,+\infty \right). \end{array}$$

The gamma prior distribution has been previously considered, e.g., in Geweke (1993) and Wang et al. (2011). For the use of an improper prior, see Fonseca et al. (2008). Since we are using a proper prior distribution for *B*, its posterior distribution is well-defined for ν > 0 (Geweke, 1993), whereas the constraint ν > 2 is required when using improper prior distributions.

### 3.2. Posterior Approximation

Denote the collection of parameters with **θ** = (*B*, Σ_1_, Σ_2_, ν), and let **Y** = (*Y*_1_, …, *Y*_*T*_) be the collection of all observed networks. The likelihood of the model in Equation (1) is

(7) $$\begin{array}{l} P\left(\text{Y}|\theta \right)=\overset{T}{\underset{t=1}{\prod }}\frac{\Gamma_{n}(\frac{\nu +2n-1}{2})}{\pi^{\frac{n^{2}}{2}}\Gamma_{n}\left(\frac{\nu +n-1}{2}\right)}|\Sigma_{1}{|}^{-\frac{n}{2}}|\Sigma_{2}{|}^{-\frac{n}{2}}|I_{n}\\ \;+\Sigma_{1}^{-1}\left(Y_{t}-B\right)\Sigma_{2}^{-1}{\left(Y_{t}-B\right)}^{\prime }{|}^{-\frac{\nu +2n-1}{2}}. \end{array}$$

Since the joint posterior distribution implied by the prior assumptions in Equations (3)–(6) and the likelihood in Equation (7) is not tractable, we follow a data augmentation approach. We exploit the representation of the matrix *t* distribution as a scale mixture of matrix normal distributions, with Wishart mixing distribution (Thompson et al., 2020). From Theorem 4.3.1 in Gupta and Nagar (1999), if $S\sim W_{p}\left(\Sigma_{1}^{-1},\nu +p-1\right)$ and $X|S\sim N_{p,m}\left(M,S^{-1},\Sigma_{2}\right)$, then *X*~*t*_*p, m*_(ν, *M*, Σ_1_, Σ_2_). Following Gelman et al. (2014) parametrization of the inverse Wishart, we obtain the equivalent representation $W=S^{-1}\sim {IW}_{p}\left(\Sigma_{1},\nu +p-1\right)$ and $X|W\sim N_{p,m}\left(M,W,\Sigma_{2}\right)$. We apply this result to *Y*_*t*_~*t*_*n, n*_(ν, *B*, Σ_1_, Σ_2_) and obtain the complete data likelihood

(8) $$\begin{array}{ll} P(Y,W|\theta ) & =\prod_{t=1}^{T}[{\left(2\pi \right)}^{-\frac{n^{2}}{2}}|W_{t}{|}^{-\frac{n}{2}}|\Sigma_{2}{|}^{-\frac{n}{2}}\;\\ & \exp\left(-\text{​}\frac{1}{2}\mathrm{tr}(\Sigma_{2}^{-1}(Y_{t}-B{)}^{\prime }W_{t}^{-1}(Y_{t}-B))\right)\\ & \cdot \frac{|\Sigma_{1}{|}^{\frac{\nu +n-1}{2}}}{2^{\frac{(\nu +n-1)n}{2}}\text{​}\Gamma_{n}(\frac{\nu +n-1}{2})}|W_{t}{|}^{-\frac{\nu +2n}{2}}\exp\text{​}\left(-\text{​}\frac{1}{2}\mathrm{tr}(\Sigma_{1}\text{​}W_{t}^{-1})\text{​}\right)\text{​}], \end{array}$$

where **W** = (*W*_1_, …, *W*_*T*_) is the collection of auxiliary variables, with $W_{t}\sim {IW}_{n}\left(\Sigma_{1},\nu +n-1\right)$.

The data augmentation approach combined with our prior assumptions allows us to derive analytically the full conditional distributions of *B*, Σ_1_, Σ_2_, and **W**. Since the joint posterior distribution is not tractable, we implement an MCMC approach based on a Gibbs sampling algorithm that iterates over the following steps:

1. Draw (ν, **W**) from the joint posterior distribution *P*(ν, **W**|**Y**, *B*, Σ_1_, Σ_2_) with a collapsed-Gibbs step that first samples ν ~ *P*(ν|**Y**, *B*, Σ_1_, Σ_2_) and then **W** ~ *P*(**W**|**Y**, *B*, Σ_1_, Σ_2_, ν).
2. Draw vec(*B*) from the multivariate normal distribution *P*(vec(*B*)|**Y**, **W**, Σ_1_, Σ_2_).
3. Draw Σ_1_ from the Wishart distribution *P*(Σ_1_|**W**, ν, γ).
4. Draw Σ_2_ from the inverse Wishart distribution *P*(Σ_2_|**Y**, *B*, **W**, γ).
5. Draw γ from the gamma distribution *P*(γ|Σ_1_, Σ_2_).

See the Appendix for further details.

### 3.3. Simulation Experiments

We study the effects of the network estimation errors on the network statistics. We set the size of the network to *n* = 70, assume the degrees of freedom parameter takes values ν = 1, 2, …, 50, and consider two experimental settings with different levels of variance in the error term *E*_*t*_: (i) low variance, with Σ_1_ = *I*_*n*_ · 3.0 and Σ_2_ = *I*_*n*_ · 1.2, and (ii) high variance, with Σ_1_ = *I*_*n*_ · 75.0 and Σ_2_ = *I*_*n*_ · 1.2. The choice of the parameter settings reflects the results obtained in the empirical application.

The adjacency matrix *A* of the network is obtained by applying the probit transformation to the elements of *B*. Following common practice in the analysis of financial connectedness [e.g., see Billio et al., 2012], we fix a threshold equal to 0.05, that is *a*_*ij*_ = 𝕀(Φ(*b*_*ij*_ < 0.05)), where *a*_*ij*_ and *b*_*ij*_ are the (*i, j*)-th elements of *A* and *B*, respectively, 𝕀(*p*) is the indicator function, taking value 1 when *p* is true and 0 otherwise, and Φ denotes the cdf of the standard normal distribution.

We focus on four measures of node centrality commonly used in network analysis (Newman, 2018, Chapter 7): out-degree, $d_{i}^{out}$, eigenvector centrality, $c_{i}^{e}$, closeness centrality, $c_{i}^{c}$, and betweenness centrality, $c_{i}^{b}$. To define these measures, we first introduce some notation. A path is a sequence of edges which joins a sequence of distinct vertices, and a node *s* is said to be reachable from node *t* if there exists a path which starts with *t* and ends with *s*.

The out-degree on node *i* is the total number of outgoing connections, that is

$$\begin{array}{l} d_{i}^{out}=\overset{n}{\underset{j=1}{\sum }}Y_{ji,t}. \end{array}$$

The eigenvector centrality describes the influence of a node in a network by accounting for the centrality of all the other nodes in its neighborhood. For each node *i*, it is defined as

(9) $$\begin{array}{r} c_{i}^{b}=\frac{1}{\lambda }\underset{v\in N_{i}}{\sum }a_{iv}c_{v}^{b}, \end{array}$$

where the score $c_{i}^{b}$ is related to the score of its neighborhood *N*_*i*_ = {*v* ∈ *V*; *a*_*iv*_ = 1} and λ is an eigenvalue of the adjacency matrix *A*. The closeness centrality accounts for connectivity patterns by indicating how easily a node can reach other nodes. For each node *i*, it is given by

(10) $$\begin{array}{r} c_{i}^{c}=\frac{n-1}{\sum_{v\in V,v\neq i}l\left(i,v\right)} \end{array}$$

where *l*(*i, v*) is the length of the shortest path between *i* and *v*. A related measure is the betweenness centrality, which indicates how relevant a node is in terms of connecting other nodes in the graph. Let *n*(*u, v*) be the number of shortest paths $P_{uv}^{*}$ from *u* to *v* and *n*_*i*_(*u, v*) be the cardinality of the set $\left\{P_{uv}^{*}:i\in P_{uv}^{*}\right\}$, that is the number of shortest paths from *u* to *v* going through the node *i*. Then the betweenness centrality for node *i* is

(11) $$\begin{array}{r} c_{i}^{b}=\underset{u\neq v,i\notin \left\{u,v\right\}}{\sum }\frac{n_{i}\left(u,v\right)/n\left(u,v\right)}{\left(n-1\right)\left(n-2\right)}. \end{array}$$

**Figure 1:**
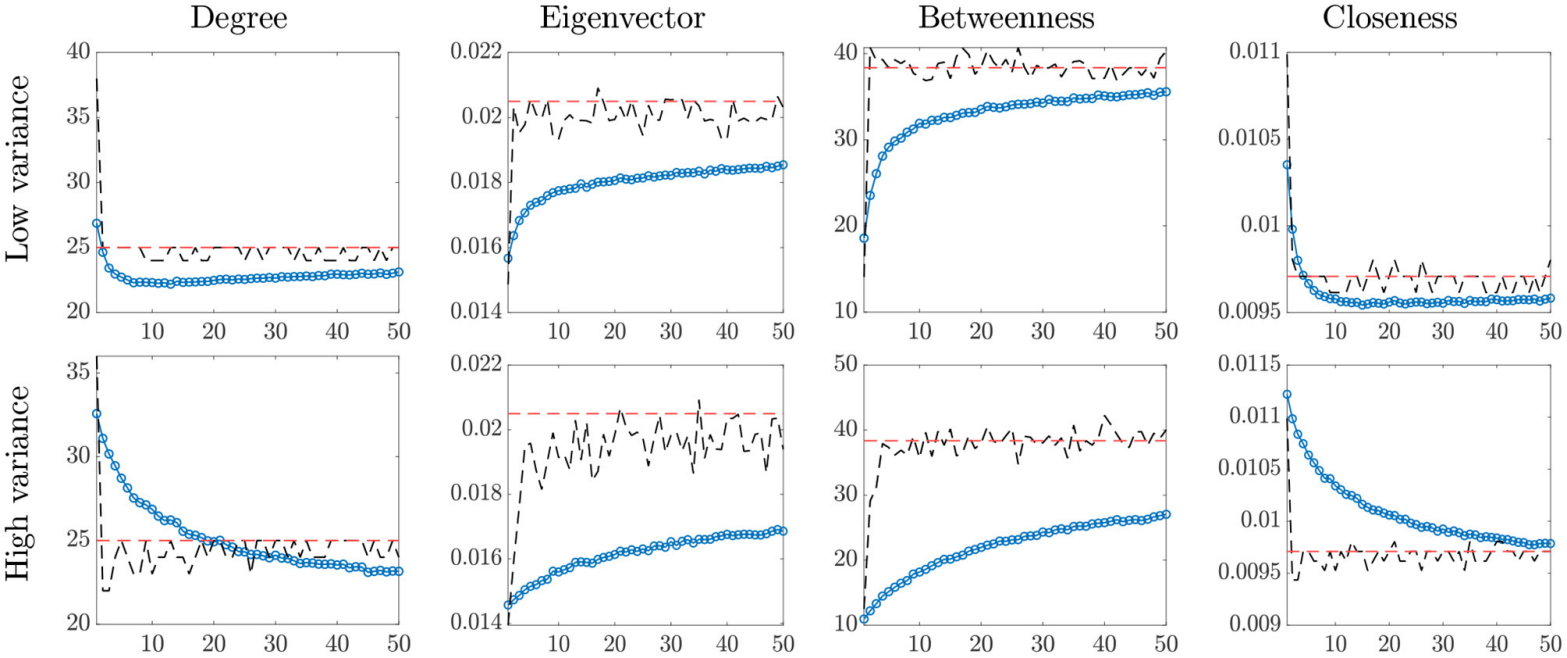
Network centrality statistics. In each plot: true value (red, dashed line) and temporal averages of the statistics on the raw data *Y*_*t*_ (blue, dotted line), and statistics based on the estimated network $\hat{B}$ (black, dashed line), for increasing values of the degrees of freedom, ν (horizontal axis).

Figure 1 shows the network statistics for the true network *A*, the estimated network based on $\hat{B}$, and the empirical averages of the statistics based on the raw data *Y*_*t*_. The main findings are summarized below:

- for heavier-tailed noise distribution (i.e., smaller ν), the bias of the empirical network statistics increases;
- the bias increases with the scale of the noise, especially for the eigenvector and betweenness centrality measures;
- as the degrees of freedom increase, the noise distribution converges to a matrix normal and the empirical averages approach the true value;
- the model proposed in Equation (1) yields correct estimates of the metrics for all values of ν, even in presence of heavy tails, with higher dispersion as the scale of the noise increases.

Overall, these findings indicate that a direct implementation of network analysis in presence of noisy measurements may lead to misguiding conclusions. This issue can be addressed by using the proposed methodology to de-noise the network data.

## 4. Empirical Analysis

In this section, we provide a description of the raw time series data and of the methodology used to extract the financial networks. Then, we present the benefits of the proposed model for computing the summary statistics most widely used in applied network analysis.

### 4.1. Data Description

We consider the stock prices of the 70 European firms with the largest market capitalization (source: Bloomberg and Eikon/Datastream). The dataset includes 28 German, 37 French, and five Italian firms, belonging to 11 GICS sectors: Communication Services (four firms), Consumer Discretionary (15 firms), Consumer Staples (six firms), Energy (two firms), Financials (11 firms), Health Care (six firms), Industrials (10 firms), Information Technology (five firms), Materials (two firms), Real Estate (three firms), Utilities (five firms), and Food and Beverages (one firm)^1^.

Data are sampled from the 4th of January 2016 to the 31st of December 2019, at weekly frequency (Friday-Friday). The period after the outbreak of the COVID-19 is excluded from the analysis due to the break induced on the network structure.

We extract the network sequence using the pairwise Granger-causality test (e.g., see Billio et al., 2012) on a rolling window of 104 weekly logarithmic returns (i.e., 2 years). The auxiliary regression used is

(12) $$\begin{array}{r} \{\begin{array}{c} x_{i,t}=\beta_{11}x_{i,t-1}+\beta_{12}x_{j,t-1}+\varepsilon_{it}\\ x_{j,t}=\beta_{21}x_{i,t-1}+\beta_{22}x_{j,t-1}+\varepsilon_{jt} \end{array} \end{array}$$

where *i, j* = 1, …, *n*, for *i* ≠ *j*. Each entry (*i, j*) of the matrix *Y*_*t*_, denoted by *Y*_*ij, t*_, is the *p*-value of the Granger test statistic. The element *Y*_*ij, t*_ represents the probability that *x*_*j, t*_ Granger-causes *x*_*i, t*_. We estimate a total of 105 adjacency matrices, for the period from the 29th of December 2017 to the 31st of December 2019^2^.

### 4.2. Results

In this section, we apply the model and inference proposed in sections 2, 3 to estimate the impact of the risk factors on the European financial network. We run the Gibbs sampler for 5,000 iterations after discarding the first 2,000 as burn-in^3^.

**Figure 2:**
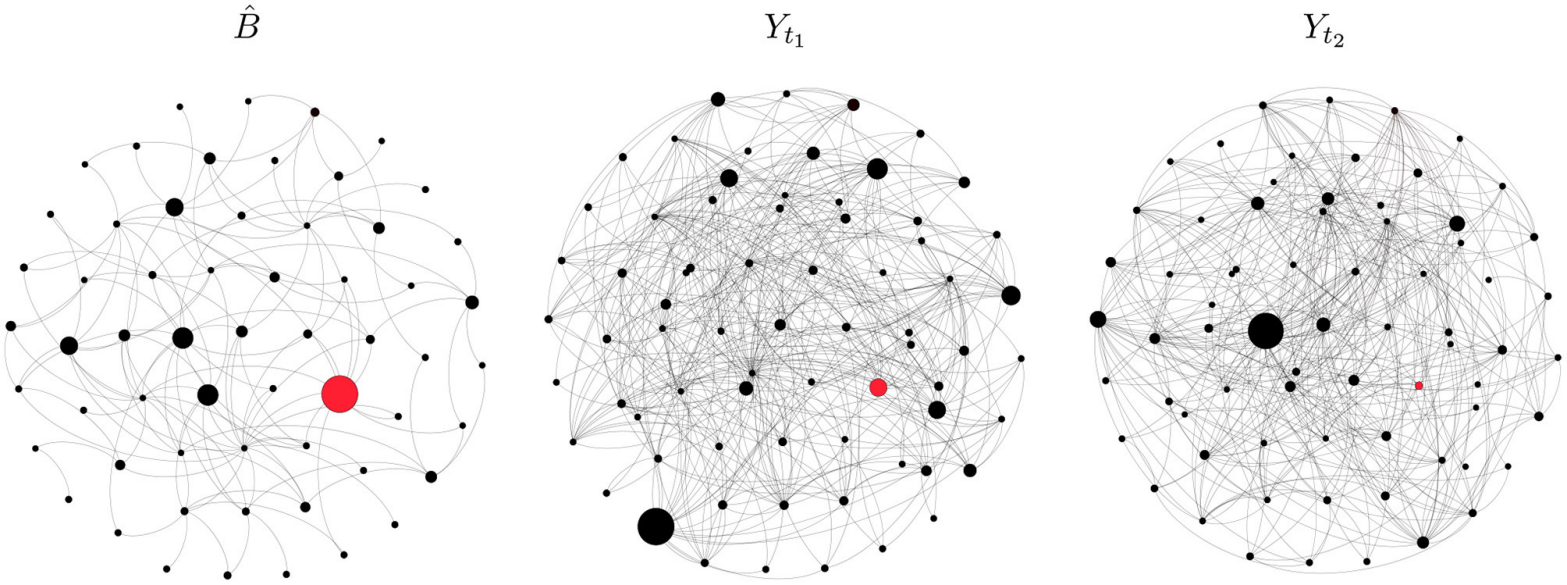
Graphical representation of the de-noised network (left) and raw networks at *t*_1_ = 1 (middle) and *t*_2_ = 105 (right). Black dots represent financial firms and gray arcs represent directed edges (clockwise orientation). The red dot stands for the most central institution according to degree centrality. In each plot, the size of a node is proportional to its out-degree.

Figure 2 shows the de-noised directed network $\hat{B}$ and two elements of the raw series. In each plot, a node represents a firm and its size is proportional to its out-degree. The red dot indicates the most central node in $\hat{B}$, that is the node with the highest out-degree in $\hat{B}$, and directed edges are represented by clockwise-oriented arcs. The node with the highest out-degree is a financial firm, whereas the most central node according to the other measures belongs to the energy sector. The largest black dot in each plot of Figure 2 represents the most degree-central institution, that is the one with highest out-degree. As shown by the position of the largest dot in the middle and right plots, the most central node varies over the sample. This supports the claim that observation contaminated by noise, if not properly filtered, may alter network analyses.

**Figure 3:**
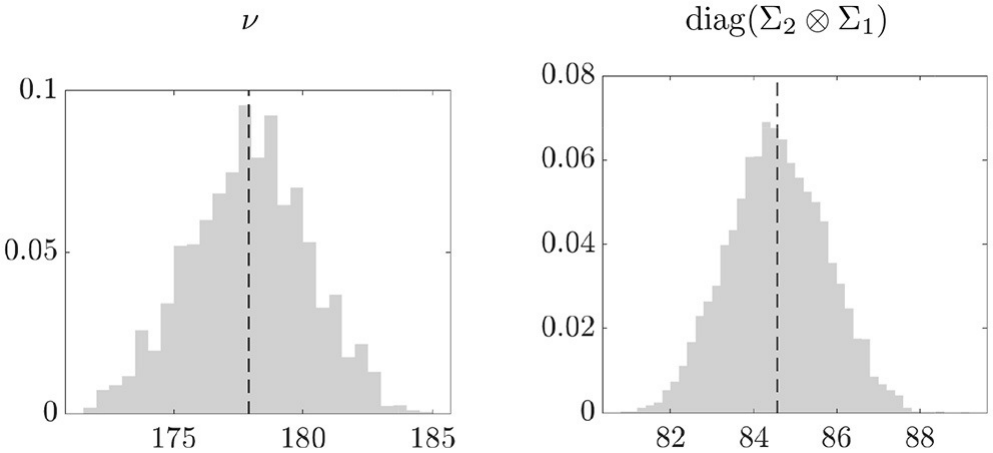
Posterior distribution (gray) and mean (black, dashed line) of the degrees of freedom (left) and of the average of diag(Σ_2_ ⊗ Σ_1_) (right).

The posterior density of the degrees of freedom in the left plot of Figure 3 suggests that the noise distribution is close to the Gaussian. Nonetheless, as shown in the simulation experiments, our approach is able to provide more accurate estimates of the network measures as compared to the empirical averages. This is particularly evident for the eigenvector centrality, where the empirical averages are sensitive to errors compatible with a *t* distribution with large degrees of freedom (see Figure 1). The right plot shows the posterior distribution of the average of the elements on the main diagonal of Σ_2_ ⊗ Σ_1_. The estimated average variance is 84.5, thus providing evidence of high variability in the network observations.

**Figure 4:**
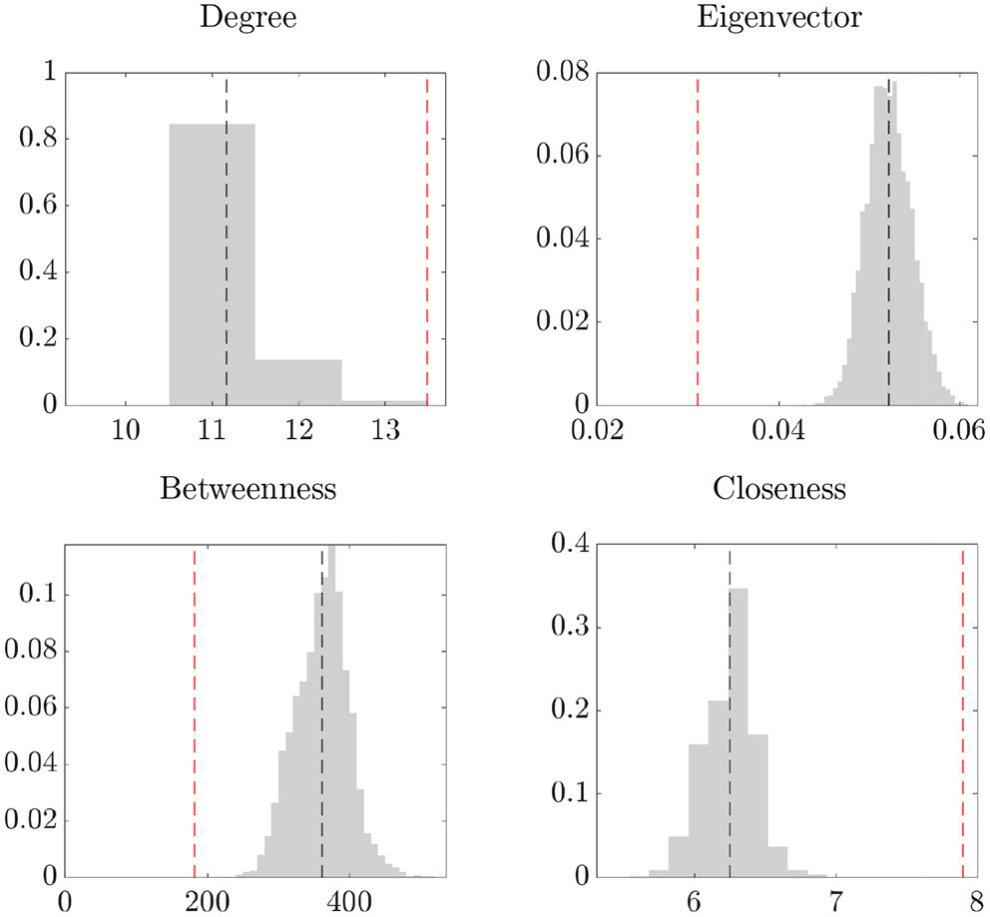
Network centrality statistics. In each plot: posterior distribution (gray) and mean (black, dashed line) of the statistics, and temporal averages of the statistics on the raw data *Y*_*t*_ (red, dashed line).

As described in section 1, the presence of noise in the data can invalidate network analyses, such as the identification of the most central institution. Motivated by this fact, we assess the importance of de-noising the network data by computing the network centrality measures on the raw data and on the de-noised network obtained using the method in section 2. The results are shown in Figure 4, which reports the posterior distribution of the centrality measures computed on the de-noised network (gray, with the black line representing the posterior mean) and the temporal average of the statistics computed on the raw data (red line). We find that all centrality measures of the de-noised data differ from the temporal average of the raw data; in particular, the eigenvector and betweenness centrality based on the raw data are underestimated, while the closeness centrality is overestimated. Overall, these findings provide evidence that the presence of noise in network data may jeopardize the validity of the ensuing analyses of the network structure.

## 5. Conclusions

A common, though often neglected, aspect of network analysis is that observations for networks might come with errors from different sources, such as estimation and measurement errors. We show that noise may invalidate the study of the network topology, such as the measurement of node centrality.

We have introduced a new matrix-variate regression framework that allows for heavy-tailed matrix-variate *t* errors to address this issue. The model is applied to filter out the noise from network data as a preliminary step before investigating the connectedness structure. In the presence of heavy-tailed error distributions or big scales of the variance of the noise, the proposed approach has superior performance compared to the temporal averages of the network statistics. Finally, we have applied the model to a sequence of estimated networks among European firms and find evidence of large error variance that affects the centrality measures.

More generally, our approach can be implemented to obtain robust network inference or fit benchmark random network models, such as those proposed by Erdőrigos-Rény and Albert-Barabási, thus representing a valuable tool for researchers investigating networks.

In this paper, we focus on the case where all the noisy observations contain the same set of nodes, meaning that there is no uncertainty on the network nodes. An interesting extension would be to consider observed networks having different sizes. We leave this for future research.

## Data Availability Statement

The original contributions presented in the study are included in the article/Supplementary Material, further inquiries can be directed to the corresponding author/s.

## Author Contributions

All authors listed have made a substantial, direct and intellectual contribution to the work, and approved it for publication.

## Conflict of Interest

The authors declare that the research was conducted in the absence of any commercial or financial relationships that could be construed as a potential conflict of interest.

## Footnotes

^1^The list of the firms, the countries, and the information about their GICS sectors and industries are available upon request to the Authors.

^2^The estimation algorithm has been parallelized and implemented in MATLAB on the High Performance Computing (HPC) cluster at Ca' Foscari University. Each node has two CPUs Intel Xeon with 20 cores 2.4 GHz and 768 GB of RAM.

^3^The total computing time for the empirical application is about 22 h with code written in MATLAB and run on an Intel Xeon with 20 cores 2.4 GHz and 768 GB of RAM.
