# Supplementary material for: A Matrix-Variate t Model for Networks
Source: Front Artif Intell. 2021 May 13;4:674166. doi: 10.3389/frai.2021.674166 (PMC8158295; doi:10.3389/frai.2021.674166)
Supplement: Supplementary file 1 [file Data_Sheet_1.pdf]

Appendix for "A matrix-variate t model for networks"

Monica Billio[*] Roberto Casarin[†] Michele Costola[‡] Matteo Iacopini[§]

April 30, 2021

A Full conditional distributions

Sampling B The coefficient matrix is drawn from the posterior full conditional distribution

$$
\begin{aligned}
&P(B|\mathbf{Y},\mathbf{W},\Sigma_1,\Sigma_2) \propto P(B)P(\mathbf{Y},\mathbf{W}|B,\Sigma_1,\Sigma_2) \\
&\propto \exp\Big(-\frac{1}{2}\operatorname{tr}\big(\operatorname{vec}(B)'(\Omega_2\otimes\Omega_1)^{-1}\operatorname{vec}(B)\big) - \frac{1}{2}\sum_{t=1}^{T}\operatorname{tr}\big(\operatorname{vec}(Y_t-B)'(\Sigma_2\otimes W_t)^{-1}\operatorname{vec}(Y_t-B)\big)\Big) \\
&\propto \exp\Big(-\frac{1}{2}\operatorname{tr}\big(\operatorname{vec}(B)'\big[(\Omega_2\otimes\Omega_1)^{-1}+\sum_{t=1}^{T}(\Sigma_2\otimes W_t)^{-1}\big]\operatorname{vec}(B)-2\sum_{t=1}^{T}\operatorname{vec}(Y_t)'(\Sigma_2\otimes W_t)^{-1}\operatorname{vec}(B)\big)\Big) \\
&\propto \exp\Big(-\frac{1}{2}\operatorname{tr}\big(\operatorname{vec}(B)'\overline{\Omega}^{-1}\operatorname{vec}(B) - 2\operatorname{vec}(\overline{M})'\overline{\Omega}^{-1}\operatorname{vec}(B)\big)\Big),
\end{aligned}
$$

where

$$
\overline{\Omega} = \Big[(\Omega_2\otimes\Omega_1)^{-1} + \sum_{t=1}^{T}(\Sigma_2\otimes W_t)^{-1}\Big]^{-1}, \qquad \operatorname{vec}(\overline{M}) = \overline{\Omega}\sum_{t=1}^{T}(\Sigma_2\otimes W_t)^{-1}\operatorname{vec}(Y_t)
$$

meaning that $\operatorname{vec}(B) \sim \mathcal{N}_{n^2}(\operatorname{vec}(\overline{M}),\overline{\Omega})$.

Sampling W_t The auxiliary covariance matrices W_t, for $t = 1,\ldots,T$, are drawn from the posterior full conditional distribution

$$
\begin{aligned}
P(W_t|\mathbf{Y},B,\Sigma_1,\Sigma_2,\nu) &\propto P(\mathbf{Y},\mathbf{W}|B,\Sigma_1,\Sigma_2,\nu) \\
&\propto |W_t|^{-\frac{n+\nu+n+1+n-1}{2}} \exp\Big(-\frac{1}{2}\operatorname{tr}\big((Y_t-B)\Sigma_2^{-1}(Y_t-B)'W_t^{-1}\big) - \frac{1}{2}\operatorname{tr}\big(\Sigma_1 W_t^{-1}\big)\Big)
\end{aligned}
$$

[*]Ca' Foscari University of Venice, Email: billio@unive.it. (corresponding author)
[†]Ca' Foscari University of Venice, Email: r.casarin@unive.it.
[‡]Ca' Foscari University of Venice, Email: michele.costola@unive.it.
[§]Vrije Universiteit Amsterdam and Tinbergen Institute, Email: m.iacopini@vu.nl.

$$\propto \big|W_t\big|^{-\frac{(\nu+2n-1)+n+1}{2}} \exp\Big(-\frac{1}{2}\operatorname{tr}\big(\Sigma_1 + (Y_t - B)\Sigma_2^{-1}(Y_t - B)'W_t^{-1}\big)\Big)$$
$$\propto \mathcal{IW}_n(\overline{W}, \overline{\nu})$$

where

$$\overline{W} = \Sigma_1 + (Y_t - B)\Sigma_2^{-1}(Y_t - B)' \qquad \overline{\nu} = \nu + 2n - 1.$$

Sampling Σ_1 Given a Wishart prior, the posterior full conditional distribution for Σ_1 is conjugate. Using the parametrization $\Sigma_1 \sim \mathcal{W}_n(\gamma^{-1}\Psi_1^{-1}, \kappa_1)$, one gets

$$\begin{aligned}
P(\Sigma_1|\mathbf{W}, \gamma, \nu) &\propto P(\Sigma_1|\gamma)P(\mathbf{W}|\Sigma_1, \nu)\\
&\propto \big|\Sigma_1\big|^{\frac{\kappa_1-n-1}{2}} \exp\Big(-\frac{1}{2}\operatorname{tr}\big(\gamma\Psi_1\Sigma_1\big)\Big)\prod_{t=1}^{T}\big|\Sigma_1\big|^{\frac{\nu+n-1}{2}} \exp\Big(-\frac{1}{2}\operatorname{tr}\big(\Sigma_1 W_t^{-1}\big)\Big)\\
&\propto \big|\Sigma_1\big|^{\frac{(\kappa_1+T(\nu+n-1))-n-1}{2}} \exp\Big(-\frac{1}{2}\operatorname{tr}\big(\gamma\Psi_1\Sigma_1 + \sum_{t=1}^{T}[W_t^{-1}]\Sigma_1\big)\Big)\\
&\propto \mathcal{W}_n(\overline{\Psi}_1, \overline{\kappa}_1)
\end{aligned}$$

where

$$\overline{\Psi}_1 = \big(\gamma\Psi_1 + \sum_{t=1}^{T} W_t^{-1}\big)^{-1} \qquad \overline{\kappa}_1 = \kappa_1 + T(\nu + n - 1).$$

Sampling Σ_2 Given an inverse Wishart prior, the posterior full conditional distribution for Σ_2 is conjugate. Using the properties of the Kronecker product and of the vectorization and trace operators we obtain

$$\begin{aligned}
P(\Sigma_2|\mathbf{Y}, B, \mathbf{W}, \gamma) &\propto P(\Sigma_2|\gamma)P(\mathbf{Y}, \mathbf{W}|B, \Sigma_2)\\
&\propto \big|\Sigma_2\big|^{-\frac{\kappa_2+Tn+n+1}{2}} \exp\Big(-\frac{1}{2}\big(\operatorname{tr}\big(\gamma\Psi_2\Sigma_2^{-1}\big) + \operatorname{tr}\big(\sum_{t=1}^{T}(Y_t - B)'W_t^{-1}(Y_t - B)\Sigma_2^{-1}\big)\big)\Big)\\
&\propto \big|\Sigma_2\big|^{-\frac{\kappa_2+Tn+n+1}{2}} \exp\Big(-\frac{1}{2}\operatorname{tr}\big(\gamma\Psi_2\Sigma_2^{-1} + S_2\Sigma_2^{-1}\big)\Big)\\
&\propto \mathcal{IW}_n(\overline{\Psi}_2, \overline{\kappa}_2)
\end{aligned}$$

where we defined $S_2 = \sum_{t=1}^{T}(Y_t - B)'W_t^{-1}(Y_t - B)$ and

$$\overline{\Psi}_2 = \gamma\Psi_2 + S_2 \qquad \overline{\kappa}_2 = \kappa_2 + Tn.$$

Sampling γ Using a gamma prior distribution for γ and a Wishart prior for Σ_1, with parametrization $\Sigma_1 \sim \mathcal{W}_n(\gamma^{-1}\Psi_1^{-1}, \kappa_1)$, one gets

$$\begin{aligned}
P(\gamma|\Sigma_1, \Sigma_2) &\propto P(\gamma)P(\Sigma_1|\gamma)P(\Sigma_2|\gamma) \\
&\propto \gamma^{a_\gamma - 1} e^{-\gamma/b_\gamma} \gamma^{n\frac{\kappa_1}{2}} \exp\Big(-\frac{1}{2}\operatorname{tr}\left(\gamma\Psi_1\Sigma_1\right)\Big) \gamma^{n\frac{\kappa_2}{2}} \exp\Big(-\frac{1}{2}\operatorname{tr}\left(\gamma\Psi_2\Sigma_2^{-1}\right)\Big) \\
&\propto \gamma^{a_\gamma - 1 + n\frac{\kappa_1}{2} + n\frac{\kappa_2}{2}} \exp\Big(-\gamma\Big[\frac{1}{b_\gamma} + \frac{1}{2}\operatorname{tr}\left(\Psi_1\Sigma_1\right) + \frac{1}{2}\operatorname{tr}\left(\Psi_2\Sigma_2^{-1}\right)\Big]\Big) \\
&\propto \mathcal{G}a(\overline{a}_\gamma, \overline{b}_\gamma).
\end{aligned}$$

where

$$\overline{a}_\gamma = a_\gamma + n\frac{\kappa_1}{2} + n\frac{\kappa_2}{2} \qquad \overline{b}_\gamma = \Big(\frac{1}{b_\gamma} + \frac{1}{2}\operatorname{tr}\left(\Psi_1\Sigma_1 + \Psi_2\Sigma_2^{-1}\right)\Big)^{-1}.$$

Sampling ν Combining the prior distribution and the likelihood in Eq. (6)-(7) one gets

$$\begin{aligned}
P(\nu|\mathbf{Y}, B, \Sigma_1, \Sigma_2) &\propto P(\nu)P(\mathbf{Y}|B, \Sigma_1, \Sigma_2, \nu) \\
&\propto \nu^{a_\nu - 1} e^{-\nu/b_\nu} \prod_{t=1}^{T} \frac{\Gamma_n\left(\frac{\nu+2n-1}{2}\right)}{\Gamma_n\left(\frac{\nu+n-1}{2}\right)} \left|I_n + \Sigma_1^{-1}(Y_t - B)\Sigma_2^{-1}(Y_t - B)'\right|^{-\frac{\nu+2n-1}{2}} \mathbb{I}_{(1,+\infty)}(\nu) \\
&\propto \nu^{a_\nu - 1} e^{-\nu/b_\nu} \left[\frac{\Gamma_n\left(\frac{\nu+2n-1}{2}\right)}{\Gamma_n\left(\frac{\nu+n-1}{2}\right)}\right]^T \left[\prod_{t=1}^{T} \left|I_n + \Sigma_1^{-1}(Y_t - B)\Sigma_2^{-1}(Y_t - B)'\right|\right]^{-\frac{\nu+2n-1}{2}} \mathbb{I}_{(1,+\infty)}(\nu) \\
&\propto \mathcal{TG}a(\overline{a}_\nu, \overline{b}_\nu; 1, +\infty) \left[\frac{\Gamma_n\left(\frac{\nu+2n-1}{2}\right)}{\Gamma_n\left(\frac{\nu+n-1}{2}\right)}\right]^T
\end{aligned}$$

where

$$\overline{a}_\nu = a_\nu \qquad \overline{b}_\nu = \Big[\frac{1}{b_\nu} + \frac{1}{2}\sum_{t=1}^{T} \log\Big(\left|I_n + \Sigma_1^{-1}(Y_t - B)\Sigma_2^{-1}(Y_t - B)'\right|\Big)\Big]\Big)\Big]^{-1}.$$

We sample from this distribution using an adaptive RWMH step with truncated logNormal proposal distribution (Atchadé et al., 2005).

References

Atchadé, Y. F., Rosenthal, J. S., et al. (2005). On adaptive Markov chain Monte Carlo algorithms. *Bernoulli*, 11(5):815–828.
